# The multifunctional protein CI of potyviruses plays interlinked and distinct roles in viral genome replication and intercellular movement

**DOI:** 10.1186/s12985-015-0369-2

**Published:** 2015-09-15

**Authors:** Ping Deng, Zujian Wu, Aiming Wang

**Affiliations:** College of Plant Protection, Fujian Agriculture and Forestry University, Fuzhou, Fujian 350002 P.R. China; Agriculture and Agri-Food Canada, 1391 Sandford St., London, ON N5V 4T3 Canada

## Abstract

**Background:**

The multifunctional cylindrical inclusion (CI) protein of potyviruses contains ATP binding and RNA helicase activities. As part of the viral replication complex, it assists viral genome replication, possibly by binding to RNA and unwinding the RNA duplex. It also functions in viral cell-to-cell movement, likely via the formation of conical structures at plasmodesmata (PD) and the interaction with coat protein (CP).

**Methods:**

To further understand the role of CI in the viral infection process, we employed the alanine-scanning mutagenesis approach to mutate CI in the infectious full-length cDNA clone of *Turnip mosaic virus* (TuMV) tagged by green fluorescent protein. A total of 40 double-substitutions were made at the clustered charged residues. The effect of these mutations on viral genome amplification was determined using a protoplast inoculation assay. All the mutants were also introduced into *Nicotiana benthamiana* plants to assess their cell-to-cell and long-distance movement. Three cell-to-cell movement-abolished mutants were randomly selected to determine if their mutated CI protein targets PD and interacts with CP by confocal microscopy.

**Results:**

Twenty CI mutants were replication-defective (5 abolished and 15 reduced), one produced an elevated level of viral genome in comparison with the parental virus, and the remaining 19 retained the same replication level as the parental virus. The replication-defective mutations were predominately located in the helicase domains and C-terminal region. All 15 replication-reduced mutants showed delayed or abolished cell-to-cell movement. Nine of 20 replication-competent mutants contained infection within single cells. Five of them distributed mutations within the N-terminal 100 amino acids. Most of replication-defective or cell-to-cell movement-abolished mutants failed to infect plants systemically. Analysis of three randomly selected replication-competent yet cell-to-cell movement-abolished mutants revealed that the mutated CI failed to form regular punctate structures at PD and/or to interact with CP.

**Conclusions:**

The helicase domain and C-terminal region of TuMV CI are essential for viral genome replication, and the N-terminal sequence modulates viral cell-to-cell movement. TuMV CI plays both interlinked and distinct roles in replication and intercellular movement. The ability of CI to target PD and interact with CP is associated with its functional role in viral cell-to-cell movement.

**Electronic supplementary material:**

The online version of this article (doi:10.1186/s12985-015-0369-2) contains supplementary material, which is available to authorized users.

## Background

Systemic infection by plant viruses results from the complex molecular interplay between the host plant and the invading virus [1]. To establish systemic infection, plant viruses must have the ability to generate progeny viruses in the primarily infected cell, move from therein to neighboring cells and further transport long-distance within the plant. Viral cell-to-cell or local movement mainly occurs in mesophyll and epidermal cells through plasmodesmata (PD), a specialized intercellular organelle that crosses the cell wall to establish cytoplasmic and endomembrane continuity between adjacent cells [2], whereas the phloem-dependent long-distance transport allows the virus to reach remote tissues through the vascular system [3]. Currently, it is generally accepted that viral intercellular movement is mediated by virus-encoded movement protein (MP) [4], although the number of MPs, their interactions with host cellular structure and mode of action are different from virus to virus.

Potyviruses represent the largest group of known plant viruses and include many agriculturally important viruses such as *Turnip mosaic virus* (TuMV), *Plum pox virus*, *Tobacco etch virus* (TEV), and *Soybean mosaic virus* [5]. The potyviral genome is a single positive-strand RNA and encodes a long polyprotein that is processed by three proteinases (P1, HC-Pro and NIaPro) to release 10 mature proteins [5]. A frameshift resulting from replication slippage in the P3 cistron leads to the production of an additional protein P3N-PIPO [6–8]. Although five viral proteins, i.e., the cylindrical inclusion protein (CI), the coat protein (CP), the helper component proteinase (HC-Pro), the viral genome-linked protein (VPg) and P3N-PIPO have been implicated in viral intercellular movement, CI seems to play a direct role in viral cell-to-cell movement [3, 9].

Previous studies have shown that potyviral CI is an RNA helicase [10]. As highly conserved enzymes, RNA helicases can utilize ATP to catalyze the separation of RNA duplexes and the structural rearrangement of RNA and RNA/protein complexes (ribonucleoprotein (RNP) complexes) in all aspects of RNA metabolism, from transcription, mRNA splicing and translation, RNA modification and transport, ribosome biogenesis, RNP complex assembly to mRNA degradation [11, 12]. RNA helicases are present in all eukaryotic cells as well as many bacteria and some viruses [13]. Based on sequence and structural features, RNA helicases are classified into five main groups, namely, superfamily (SF) 1 to SF5 [14]. The potyviral CI RNA helicase belongs to SF2 and contains seven highly conserved motifs, I, Ia, II, III, IV, V, and VI. Motifs Ia, III, and IV are the least conserved, whereas motif IV as well as motifs I, II, and V are the best conserved [15]. The helicase domain of the CI protein is located at the N-terminal and central region. The C-terminal region shows no homology with any known proteins. Genetic analyses conducted with a TEV full-length infectious clone tagged with the marker gene encoding b-glucuronidase (GUS) provided genetic evidence that the CI protein plays an essential role in viral genome replication and cell-to-cell movement [3]. Consistent with the genetic data, CI has been found to be present in the viral replication complex, presumably assisting viral genome amplification through its RNA binding and duplex unwinding activities [16, 17]. CI also interacts with CP in the cytoplasm [9, 18] and forms the conical structures at PD [9, 19, 20]. Recently we have shown that P3N-PIPO is a PD-located protein that modulates the targeting of CI to the PD to facilitate potyviral cell-to-cell movement [9]. The structure and function of potyviral CI have been reviewed recently [21]. Despite this progress, the mode of action by CI and its underlying mechanism in the potyviral infection process are still far from being understood.

In this study, we generated a serial of double-substitutions at the clustered charged residues of the CI protein of TuMV and analyzed the effect of these mutations on viral genome amplification, cell-to-cell movement and systemic infection. Our data support that the potyviral CI protein has essential, yet distinct roles in viral replication and intercellular movement.

## Results and discussion

### Analysis of the TuMV CI amino acid sequence and construction of alanine-scanning double-substitution mutants

**Fig. 1:**
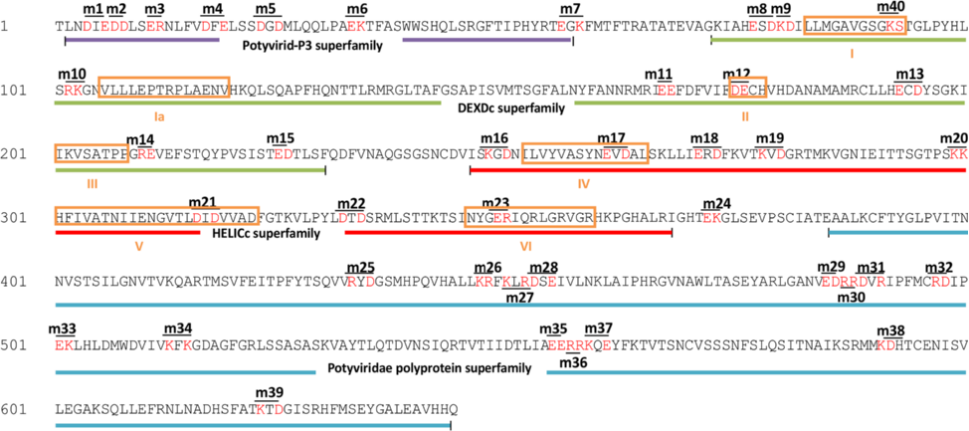
The localization of the four conserved domains of the CI protein of TuMV and the substitution residues of CI mutants. Conserved domains are determined according to NCBI conserved domain database (CDD). The Potyvirid-P3 superfamily (aa 2–57) is underlined in purple. The DEAD- like helicases superfamily (73–230) that involved in ATP-dependent RNA or DNA unwinding are underlined in green, the helicase superfamily (246–368) in red, and the potyviridae polyprotein superfamily (386–644) in blue. Amino acids substituted with alanine in this article are colored in red, and the name of mutants containing double-substitutions are indicated above or under the mutated residues. Seven motifs (I, Ia, II, III, IV, V and VI) highly conserved for the superfamily 2 of RNA helicases are indicated in orange boxes according to [15]

The CI protein of TuMV (GenBank accession # EF028235.1) was annotated using the NCBI conserved domain database (http://www.ncbi.nlm.nih.gov/cdd) [22] Four domains were identified (Fig. 1; Additional file 1: Figure S1). The N-terminal region (57 amino acids) is part of the Potyvirid-P3 superfamily domain (GenBank accession # cl16319). This region is actually not part of the P3 protein section of the *Potyviridae* polyproteins but an extension from the P3 domain to the CI region. Followed by this region is a DEXDc domain (GenBank accession # cd00046) that belongs to the DEAD-like helicases superfamily, with the predicted function in ATP-dependent RNA or DNA unwinding. This domain contains four conserved motifs, i.e., motifs I, Ia, II and III (Fig. 1). The middle region containing about 120 amino acids is a HELICc domain (GenBank accession # cd00079), which is present in a wide variety of helicases and helicase related proteins such as DEXDc-, DEAD-, and DEAH-box proteins, yeast initiation factor 4A, Ski2p, and Hepatitis C virus NS3 helicases. This domain consists of three conserved motifs including motifs IV, V and VI (Fig. 1). Since both the DEXDc and HELICc domains are conserved among helicases and are essential for helicase activity, both of them are helicase domains. The C-terminal region is the *Potyviridae* polyprotein domain (Accession No. pfam08440) that is conserved in the polyproteins of the viral *Potyviridae* taxon. Forty double-substitutions were introduced into clustered charged amino acids of the CI protein (Fig. 1). As shown, these double-mutations were designed to spread among or near the four identified domains: 7 mutants (m1 through m7) located in the Potyvirid-P3 superfamily domain, 9 (m40 and m8 through m15) in the DEXDc superfamily domain, 9 (m16 through m24) in or near the HELICc superfamily domain, and 15 (m25 through m39) in the *Potyviradae* polyprotein superfamily domain (Additional file 1: Figure S1).

### The two helicase domains and C terminal region of the CI protein of TuMV are essential for viral replication

All the forty mutants were tested for their replication ability using a protoplast inoculation assay [23]. PEG-mediated transfection was used to deliver various plasmids into *Nicotiana benthamiana* mesophyll protoplasts. Plasmid TuMV::6 K2-GFP (wild type parental virus) was used as the positive control. Plasmid TuMV::6 K2-GFP -ΔGDD mutant (ΔGDD) was used as the negative control, where three conserved amino acids (GDD^351–353^) of the RNA-dependent RNA polymerase NIb protein in TuMV::6 K2-GFP was deleted. The highly conserved GDD motif is essential for RNA polymerase activity and deletion of the GDD motif abolishes viral replication [24]. Total RNA was extracted from protoplasts 22 h post transfection (hpi) and subjected to DNase I treatment. The viral RNA was quantified by quantitative reverse transcription-polymerase chain reaction (qRT-PCR) using *Actin* transcript as a reference. These experiments were repeated at least three times. The accumulation levels of viral genomic RNA of different mutants were compared with that of the wild type parental virus and the GDD mutant (Table 1).

**Table 1:** Effects of double-substitutions on cell-to-cell movement, long-distance spread and replication

Mutants	Residues substituted	Cell-to-cell spread	Long-distance movement	Replication	Relative levels of viral RNA
CI-m1	D4, E6	-	-	+	1.08 ± 0.14
CI-m2	D7, D8	-	-	+	1.03 ± 0.18
CI-m3	E11, R12	-	-	reduced**	0.72 ± 0.08
CI-m4	D17, E19	-	-	+	1.15 ± 0.17
CI-m5	D23, D25	slow	+	+	0.93 ± 0.13
CI-m6	E33, K34	slow	+	+	1.05 ± 0.09
CI-m7	E56, K58	+	+	+	1.02 ± 0.14
CI-m8	E77, D79	+	+	+	0.93 ± 0.07
CI-m9	K80, D81	-	-	+	1.04 ± 0.14
CI-m10	R102, K103	-	-	reduced*	0.90 ± 0.03
CI-m11	E167, E168	+	+	+	0.86 ± 0.10
CI-m12	D175, E176	-	-	-	0.52 ± 0.03
CI-m13	E193, D195	slow	+	reduced*	0.79 ± 0.09
CI-m14	R210, E211	-	-	-	0.49 ± 0.09
CI-m15	E225, D226	slow	+	reduced*	0.84 ± 0.08
CI-m16	K248, D250	-	-	-	0.62 ± 0.12
CI-m17	E261, D263	slow	-	reduced*	0.81 ± 0.06
CI-m18	E271, D273	+	+	reduced**	0.82 ± 0.04
CI-m19	K278, D280	-	-	+	1.01 ± 0.18
CI-m20	K299, K300	slow	+	reduced**	0.83 ± 0.05
CI-m21	D316, D318	-	-	+	0.95 ± 0.08
CI-m22	D332, D334	slow	+	+	0.89 ± 0.11
CI-m23	E349, R350	-	-	-	0.52 ± 0.09
CI-m24	E372, K373	slow	+	+	0.87 ± 0.13
CI-m25	R433, D435	slow	+	+	0.90 ± 0.14
CI-m26	K447, R448	slow	-	reduced*	0.73 ± 0.08
CI-m27	K450, R452	-	-	reduced**	0.72 ± 0.06
CI-m28	D453, E455	-	-	reduced**	0.68 ± 0.04
CI-m29	E485, D486	+	+	+	1.06 ± 0.04
CI-m30	R487, R488	+	+	+	0.98 ± 0.15
CI-m31	D489, R491	slow	-	reduced*	0.81 ± 0.10
CI-m32	R497, D498	-	-	-	0.50 ± 0.10
CI-m33	E501, K502	-	-	+	1.02 ± 0.12
CI-m34	K513, K515	-	-	+	1.02 ± 0.16
CI-m35	E555, E556	slow	-	reduced*	0.80 ± 0.09
CI-m36	R557, R558	-	-	reduced**	0.64 ± 0.05
CI-m37	K559, E561	-	-	reduced**	0.77 ± 0.03
CI-m38	K591, D592	slow	-	reduced*	0.71 ± 0.12
CI-m39	K623, D625	slow	+	+	0.92 ± 0.08
CI-m40	K92, S93	-	-	increased*	1.34 ± 0.17
GDD		-	-	-	0.58 ± 0.09
WT		+	+	+	1

Note: +, similar to the wild type virus; −, replication or cell-to-cell movement or long distance spread was abolished; slow, reduced spread intercellularly in the inoculated leaves; reduced, replication significantly reduced; increased, replication significantly enhanced; *, *P* < 0.05; **, *P* < 0.01

Nineteen mutants (m1, m2, m4 through m9, m11, m19, m2, m22, m24, m25, m29, m30, m33, m34 and m39) produced levels of viral genomic RNA similar to the parental virus (Table 1), suggesting mutations in these mutants do not significantly affect viral genome amplification. Five mutants, i.e., m12, m14, m16, m23 and m32 showed no viral replication, indicating these mutations are lethal to TuMV. Mutations in four of these mutants were located in the two helicase domains, suggesting the helicase function is essential for TuMV replication. This is consistent with previous findings [3]. Fifteen mutants, i.e., m3, m10, m13, m15, m17, m18, m20, m26, m27, m28, m31, m35 through m38, showed significantly reduced viral genome levels in protoplasts in comparison with the wild type parental virus (Table 1). The replication-defective mutations (in 5 lethal and 15 reduced mutants) were mainly located in the two helicase domains and the C-terminal region. Only one replication-reduced mutant contains the double-mutation located in the N-terminal domain. These data suggest that the two helicase domains and the C-terminal region of the CI protein are essential for viral replication. Interestingly, we also found one mutant, i.e., m40 containing the double-mutation in the first helicase domain, showing an elevated level of viral RNA in comparison with that of the wild type virus (Table 1). The reason underlying this is not clear.

The potyviral helicase CI possesses NTP binding and hydrolysis activities and is part of the viral replication complex (VRC) [10, 16, 17]. Mutations in the conserved motifs of CI impair its RNA binding and duplex unwinding ability and compromise viral RNA replication. Alternatively, mutations that affect the interactions of CI with other viral proteins or host factors can disrupt the formation of VRC, leading to reduced viral genome amplification.

### The N terminal region of the CI protein is required for efficient cell-to-cell movement

**Fig. 2:**
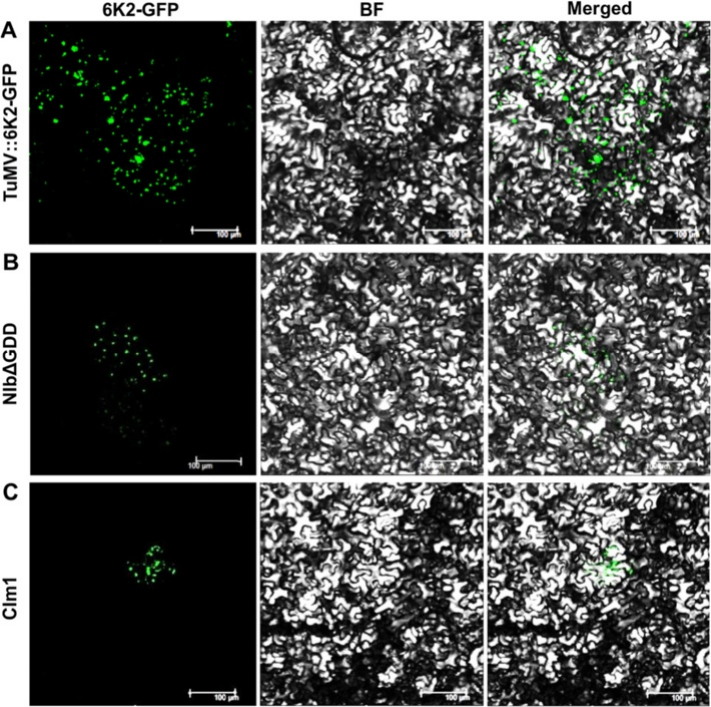
Analysis of viral cell-to-cell movement by confocal microscopy. Leaves of *Nicotiana benthamina* agro-infiltrated with the wild type parental virus TuMV::6 K2-GFP (positive control), the replication-defective mutant NIbΔGDD (negative control) and CI mutants were observed under a confocal microscope 4 days post infiltration. **a** Multicellular infection of TuMV::6 K2-GFP in the inoculated leaf. **b** Single cell expression of the replication-defective mutant NIbΔGDD (resulting from the 35S promoter). **c** Single cell infection of the TuMV::6 K2-GFP CIm1 mutant. Scale bar, 100 μm

To investigate whether the alanine substitutions of TuMV affect intercellular movement, *N. benthamiana* plants were agroinfiltrated with all 40 mutants at a very low bacterial optical density. Four days post inoculation (dpi), the inoculated leaves with the wild type parental virus (TuMV::6 K2-GFP) showed extensive large foci of GFP expression (Fig. 2). In contrast, GFP was restricted to single cells in *N. benthamiana* leaves agroinfiltrated with 20 mutants, i.e., m1 through m4, m9, m10, m12, m14, m16, m19, m21, m23, m27, m28, m32, m33, m34, m36, m37, and m40 (Table 1). The mutant m1 serves as an example (Fig. 2). Under the same conditions, 14 additional mutants, i.e., m5, m6, m13, m15, m17, m20, m22, m24 through m26, m31, m35, m38 and m39, were able to spread into neighbouring cells but showed a slow cell-to-cell movement phenotype (Table 1). Six other mutants including m7, m8, m11, m18, m29 and m30 established multicellular infection as efficiently as the wild type parental virus (Table 1).

A cross check between replication and cell-to-cell movement phenotypes revealed that all 15 replication-reduced mutants showed delayed or abolished cell-to-cell movement, suggesting an interlink between competent replication and efficient cell-to-cell movement. Of 20 replication-competent mutants, nine contained infection within single cells, demonstrating distinct roles of CI in replication and cell-to-cell movement. Moreover, based on their replication ability, the 20 cell-to-cell movement-abolished mutants may be divided into three groups. The first group (m10, m12, m14, m23 and m32) are lethal mutants. The GFP expression in the single cells actually resulted from the 35S-drected transcription of the mutated viral genome. Therefore, it is not surprising that no intercellular movement was observed for these mutants. The second group including m3, m10, m27, m28, m36, and m37 showed reduced ability to replicate in protoplasts (Table 1). For this group of mutants, viral cell-to-cell movement deficiency is apparently associated with the reduced viral genome amplification capacity. It is possible that mutations in these mutants disrupt the helicase activities that are required for viral replication as well as for viral movement [3]. P3N-PIPO, the frameshift gene product in the P3 cistron, behaves like a dedicated movement protein [9] and the replication-dependent RNA polymerase slippage is a possible mechanism for the production of P3N-PIPO [7, 8]. Since the slippage efficiency is low, the production of P3N-PIPO would require active RNA replication. Therefore, reduced viral genome amplification could lead to the cell-to-cell movement defective phenotype. The remaining 9 mutants (m1, m2, m4, m9, m19, m21, m33, m34, and m40) constitute of the last group. Mutants in this group retained their regular replication ability.

The mutations in 5 of 9 cell-to-cell movement-abolished mutants (m1, m2, m4, m9 and m40) are located within the N-terminal 100 amino acids, whereas those in the 4 remaining cell-to-cell movement-abolished mutants (m19, m21, m33 and m34) are present in the long central and C-terminal regions (Fig. 1), suggesting that the very N-terminal region is particularly important for viral cell-to-cell movement. This is consistent with previous observation that TEV CI has dual functions [3, 25]. Taken together these data clearly suggest that CI plays both interlinked and distinct roles in viral genome replication and viral intercellular movement.

### Viral long distance transport requires efficient cell-to-cell movement

**Fig. 3:**
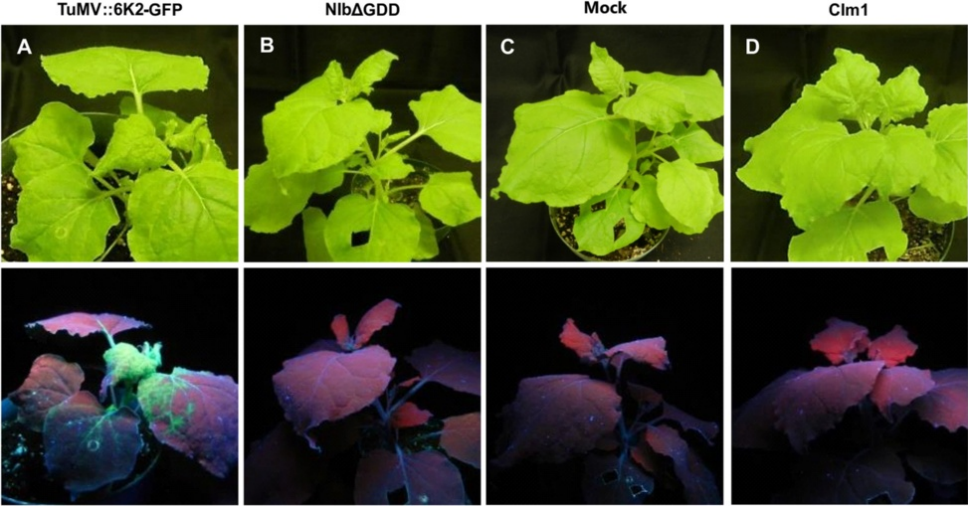
Viral long-distance movement. *Nicotiana benthamiana* plants were agro-infiltrated with the wild type parental virus TuMV::6 K2-GFP (**a**), the replication-defective mutant NIbΔGDD (**b**), mock (agrobacteria only) (**c**) and the CI mutant m1 (**d**). Photos were taken 15 days post infiltration. Top panels, photos taken under regular light; bottom panels, photos taken under UV light. The wild type parental virus TuMV::6 K2-GFP induced symptoms on new emerging leaves. Under UV light, strong GFP fluorescence was evident. No symptoms or GFP fluorescence was observed on plants agro-infiltrated with the replication-defective mutant NIbΔGDD, mock, and CIm1

Systemic infection by the 40 mutants was monitored by visualization of the green fluorescence of newly emerging leaves 15 days post inoculation followed by qRT-PCR. It was found that all the mutants whose cell-to-cell movement was not affected established systemic infection like the wild type virus (Table 1). Most of replication-defective or strict cell-to-cell movement defective mutants failed to infect plants systemically (Table 1). Under UV light, the green fluorescence was not observed in newly emerging leaves of plants inoculated with such kind of mutants (Fig. 3). The absence of the virus in the newly emerging leaves was confirmed by qRT-PCR. Taken together these data suggest that viral long distance transport requires efficient cell-to-cell movement and competent replication.

### Analysis of three cell-to-cell movement defective mutants

**Fig. 4:**
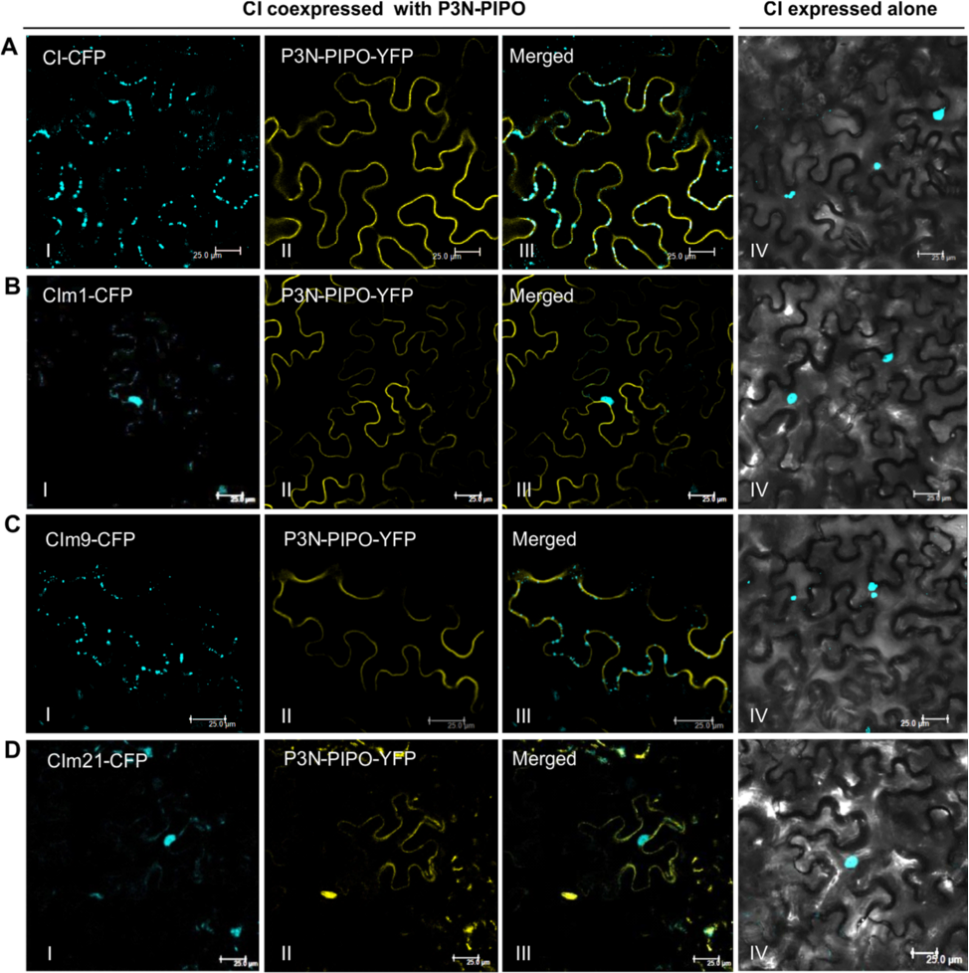
Subcellular localization of TuMV CI mutants in *Nicotiana benthamiana* leaf cells. CI from the parental virus (**a**), CIm1 (**b**), CIm9 (**c**), and CIm21(**d**) was coexpressed with P3N-PIPO (panels I to III) or expressed alone (panel IV). Scale bar, 25 μm

To further understand why these CI mutations abolish cell-to-cell movement, we randomly selected three mutants, m1, m9 and m21, for analysis. Mutations of m1 and m9 are located in the N-terminus of CI that belongs to the Potyvirid-P3 superfamily and the double-substitution in m21 is present in the central region of CI that belongs to the HELICc superfamily. Previously, we have shown that potyviral CI forms punctate structures at PD and P3N-PIPO mediates the targeting of CI to PD [9]. To test if the CI mutants can target PD, wild type CI and CI mutants were expressed alone or coexpressed with P3N-PIPO in leaves of *N. benthamiana*. Consistent with our previous data [9], the wild type CI aggregated in the cell when expressed alone but formed punctate structure at PD along the cell wall (Fig. 4a). CIm9 showed the similar subcellular localization patterns (Fig. 4c). However, when coexpressed with P3N-PIPO, CIm1 and CIm21 formed aggregates in the cell and produced very weak fluorescence signals along the cell wall (Fig. 4b, d). Since the targeting of CI to PD is required for potyviral intercellular movement, mutations in the mutants CIm1 and m21 severely inhibited the mutated form of CI to form punctate structures along the cell wall, leading to the cell-to-cell movement defective phenotype. This finding is in agreement with the observation that the TEV CI double-mutation mutants DD3,4AA or KK101,102(AA) arrests viral cell-to-cell movement and either of mutated forms of CI fails to target to PD in the presence of P3N-PIPO [3, 9]. In the case of CIm9, obviously a different mechanism is involved.

**Fig. 5:**
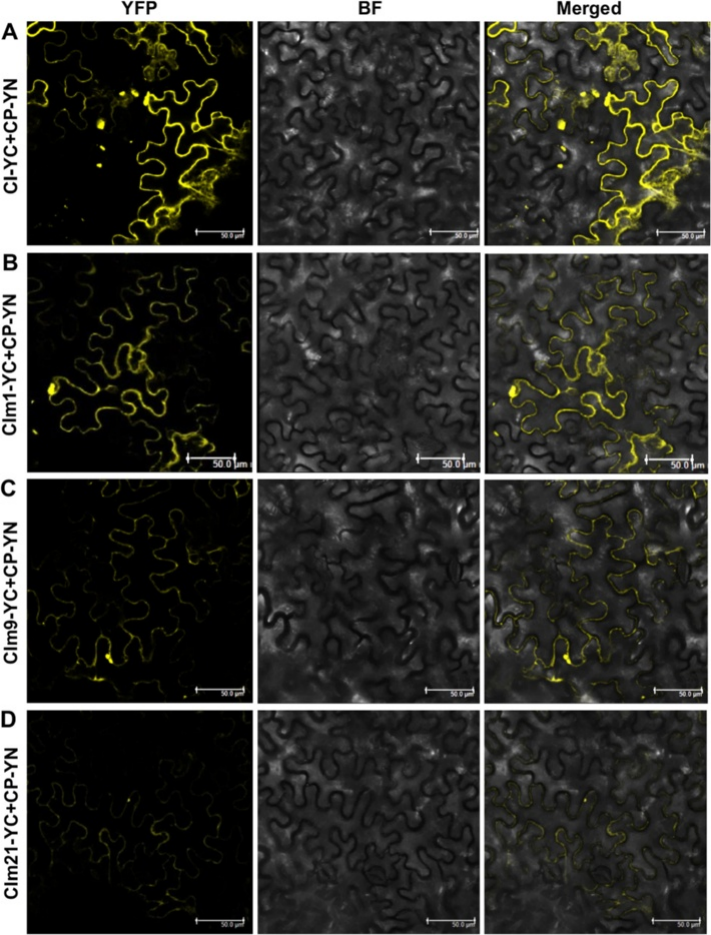
BiFC assay of interactions between CI mutants and CP. Interaction assays were conducted in leaf epidermal cells of *Nicotiana benthamiana*. Strong interactions were observed between the wild type CI and CP (**a**), and between CIm1 and CP (**b**), whereas very weak interactions were detected between CIm9 and CP (**c**), and between CIm21 and CP (**d**). Scale bar, 50 μm

Potyviral CI interacts with CP [17, 18] and is physically attached to virions [26]. It has been suggested that the CP-CI interaction is essential for viral particles to pass through PD [8]. We thus determined if these CI mutants interact with CP. Our BIFC assay revealed a strong interaction between the wild type CI and CP (Fig. 5a) as well as between CIm1 and CP (Fig. 5b). In contrast, a very weak interaction was found between CIm9 and CP (Fig. 5c) or between CIm21 and CP (Fig. 5d). Based on these data, we speculate that mutations in mutants CIm9 and CIm21 may compromise viral cell-to-cell movement though disrupting CP-CI interactions. To our best of knowledge, this is first report showing that the defect in cell-to-cell movement is linked to the CP-CI interaction.

## Conclusions

In summary, this research provided solid genetic evidence that the helicase domain and C-terminal region of TuMV CI are essential for viral genome replication, and the N-terminal sequence modulates viral cell-to-cell movement. Our data also showed that most replication-defective mutations affect viral cell-to-cell movement, and viral long-distance transport requires competent replication and efficient cell-to-cell movement. We also identified some cell-to-cell movement-abolished mutants that maintain their regular replication ability. Together these data suggest that TuMV CI plays both interlinked and distinct roles in viral replication and intercellular movement. In addition, we discovered that the ability of CI to target PD and interact with CP is associated with its functional role in viral cell-to-cell movement. Results from this research shed new insights into the functional role of CI in the potyviral infection process.

## Materials and methods

### TuMV CI sequence analysis

The TuMV genome sequence was retrieved from GenBank (http://www.ncbi.nlm.nih.gov/Genbank/) with accession numbers EF028235.1. CI protein domain families automatically generated from the NCBI conserved domain database (http://www.ncbi.nlm.nih.gov/cdd). The Potyvirid-P3 superfamily domain (Accession No. cl16319) is part of the P3 protein section of the *Potyviridae* polyproteins. The function is not known except that the protein is essential to viral survival. The DEXDc domain (Accession No. cd00046) belongs to the DEAD-like helicases superfamily, involves in ATP-dependent RNA or DNA unwinding. This domain contains the ATP-binding region. The HELICc domain (Accession No. cd00079) belongs to the helicase superfamily including DEXDc-, DEAD-, and DEAH-box proteins, yeast initiation factor 4A, Ski2p, and Hepatitis C virus NS3 helicases and is present in a wide variety of helicases and helicase related proteins. The *Potyviridae* polyprotein domain (Accession No. pfam08440) is found in polyproteins of the viral *Potyviridae* taxon.

### Alanine-scanning mutagenesis of the TuMV CI protein

The recombinant TuMV infectious clone TuMV::6 K2-GFP containing a full-length TuMV cDNA with an additional copy of 6 K2 fused to GFP was described previously [27]. To facilitate a genetic analysis of the TuMV CI protein involved in viral replication and movement, a series of substitutions of Alanine codons for clustered charged residues (D, E, K, R) in the CI coding region of TuMV::6 K2-GFP (the parental virus) were constructed. Site-directed scanning offered a rapid way to examine the role of individual charged residues for protein function [28]. Alanine was chosen to use for its simplest molecular structure and chemically inert that usually does not disturb the polypeptide chain.

Forty mutants were designed based on the alanine-scanning mutagenesis method. Clustered charged amino acids (R, K, E, D) of the CI protein indicated in Fig. 1 were substituted by alanine codons (GCC, GCT, GCA or GCG). The NIb-GDD conserved motif was deleted as a replication negative control.

A CI-containing 5274-bp cDNA fragment between *Sna*BI and *Mlu*I restriction sites (which are unique to the parental virus TuMV::6 K2-GFP) was amplified using Phusion® High-Fidelity DNA Polymerase (New England BioLabs). The PCR product was then ligated into the pCR-Blunt vector (Invitrogen). The sequence of the amplified fragment in the intermediate plasmid was confirmed by DNA sequencing, which served as the template for the introduction of designed substitutions into CI with specific primer pairs using QuikChange Site directed-mutagenesis kit (Stratagene). Upon confirmation by DNA sequencing, the mutated form of CI in the intermediate plasmids was double-digested by restriction enzymes *Sna*BI and *Mlu*I and ligated into the corresponding sites of the parental virus to generate CI mutants. DNA sequencing was performed again to confirm correct sequence.

### Protoplast isolation and transfection

Mesophyll protoplast isolation from four-week old healthy *N. benthamiana* plants were essentially performed following a published protocol [23] and subsequent PEG-mediated transfection were carried out as described previously [29]. Plasmid DNAs were extracted using the EndoFree Plasmid Maxi Kit (Qiagen) according to the manufacturer’s instructions. 2–5 × 10^5^ isolated mesophyll protoplasts were transfected with a total of 40 μg of wild type or mutant infectious clone plasmids and incubated under constant light.

### qRT-PCR

Protoplasts were harvested by centrifugation at 100Xg for two min at room temperature about 22 h post transfection. Total RNA was isolated by use of RNeasy Plant Mini Kit (Qiagen) following the supplier’s instruction. For first-strand cDNA synthesis, one μg RNA was pretreated by DNase I (Invitrogen) as a template, Superscript III reverse transcriptase and an oligo(dT) 12-18 primer(Invitrogen) were used following the manufacturer’s instruction. For real-time PCR, primer pairs TuCP-F (5′-GGCACTCAAGAAAGGCAAGG-3′) and TuCP-R (5′-CTCCGTCAGTTCGTAATCAGC-3′) were used for detection of viral genomic RNA, and the primers NbActin-F (5′-GGGATGTGAAGGAGAAGTTGGC-3′) and NbActin-R (5′-ATCAGCAATGCCCGGGAACA-3′) for the reference gene *Actin* in *N. benthamiana* were used for normalization. qRT-PCR was performed using SsoFast EvaGreen Supermix (Bio-Rad) and CFX96 real-time PCR system (Bio-Rad). The relative gene expression was calculated by Bio-Rad CFX Manager software. All the experiments were performed with at least three independent biological replicates.

### *Agrobacterium*-mediated transient expression

Plasmids containing the full-length cDNA infectious clone of the wild type parental virus or CI mutants were transformed into the *Agrobacterium tumefaciens* strain GV3101 by electroporation. Agrobacterial strains harboring plasmids containing wild type TuMV CI, P3N-PIPO and CP fused with proper fluorescence protein fusions were described previously [9]. Agrobacterial cultures were grown overnight at 28 °C on Luria-Bertani agar containing three antibiotics (50 μg/mL of rifampicin, 25 μg/mL of gentamicin and 50 μg/mL of kanamycin). Agrobacteria were harvested by centrifugation, resuspended in the infiltration buffer (10 mM MES, pH 5.6, 10 mM MgCl2, and 100uM acetosyringone) and incubated at room temperature for around 2 h before infiltrated into the lower epidermal surface of *N. benthamiana* by 1 ml syringe without needle. The agrobacterial optical density at 600 nm (OD600) was adjusted to 0.02–0.04 for cell-to-cell movement experiments and 0.4–0.6 for systemic movement observation. Expression of fluorescence protein was observed by confocal microscopy 4 days post inoculation (dpi), or under UV light 2 weeks later. *Nicotiana benthamiana* plants were grown in a growth room at 22–24 °C under a 16-h light/8-h dark cycle.

### Confocal microscopy

Confocal microscopy was performed essentially as described [30, 31]. Plant tissues and protoplasts were imaged at room temperature using a Leica TCS SP2 inverted confocal microscope. Individual cells were observed with a 10X dry objective for leaves and a 63X water immersion objective for protoplasts. Fluorescent signals were excited with an argon-krypton laser. Images were captured digitally and handled using the Leica LCS software. Postacquisition image processing was done with Adobe Photoshop software.

## Additional file

Additional file 1:
**Figure S1.** Schematic representation of the genome of the TuMV parental virus containing 6 K2-GFP (TuMV::6 K2-GFP), and the distribution of mutations in the four conserved domains of the CI protein. (TIFF 250 kb)

## Abbreviations

TuMV: Turnip mosaic virus
CI: Cylindrical inclusion protein
CP: Coat protein
MP: Movement protein
PD: Plasmodesmata
GFP: Green fluorescent protein
qRT-PCR: Quantitative reverse transcription polymerase chain reaction
