# Supplementary figures and images for: The multifunctional protein CI of potyviruses plays interlinked and distinct roles in viral genome replication and intercellular movement
Source: Virol J. 2015 Sep 15;12:141. doi: 10.1186/s12985-015-0369-2 (PMC4572616; doi:10.1186/s12985-015-0369-2)

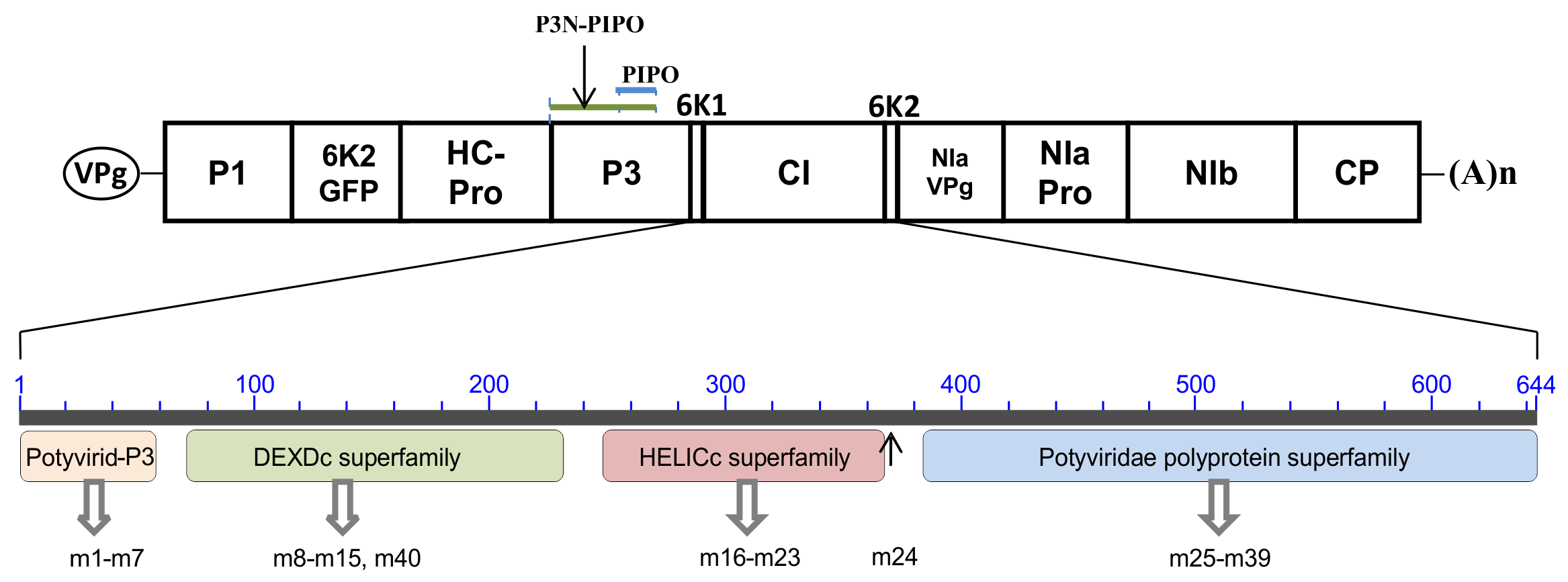

Supplement: Additional file 1: — Figure S1. Schematic representation of the genome of the TuMV parental virus containing 6 K2-GFP (TuMV::6 K2-GFP), and the distribution of mutations in the four conserved domains of the CI protein. (TIFF 250 kb) [file 12985_2015_369_MOESM1_ESM.tif]
